# The relationship between flow experience and teaching well-being of university music teachers: The sequential mediating effect of work passion and work engagement

**DOI:** 10.3389/fpsyg.2022.989386

**Published:** 2022-09-26

**Authors:** Xiaoxiao Wang

**Affiliations:** School of Music, Nanjing Normal University Taizhou College, Taizhou, China

**Keywords:** university music teachers, flow experience, teaching well-being, work passion, work engagement

## Abstract

**Objective:**

The purpose of this study was to investigate the relationship between work passion and work engagement among university music teachers in flow experience and teaching well-being.

**Methods:**

Three hundred forty-three university music teachers were tested by using the Flow State Scale, Teacher Well-Being Scale, Work Passion Scale, and Work Engagement Scale.

**Results:**

University music teachers’ flow experience can predict teaching well-being (*β* = 0.248, *p* < 0.001). University music teachers’ flow experience has an indirect impact on teaching well-being through work passion (*β* = 0.257, *p* = 0.005), and university music teachers’ flow experience has an indirect impact on teaching well-being through work engagement (*β* = 0.144, *p* = 0.018). In addition, work passion and work engagement play a chain-mediating role between university music teachers’ flow experience and teaching well-being (*β* = 0.134, *p* = 0.001).

**Conclusion:**

Work passion and work engagement play a sequential mediating role between university music teachers’ flow experience and teaching well-being.

## Introduction

Work can help individuals build good social relations, cultivate a sense of identity, and provide individuals with opportunities to contribute to social development (Savickas, 2005; Blustein, 2006). Therefore, work can be used as a source of personal well-being, achievement, and satisfaction (Myers and Diener, 1995; Angner, 2010; Kahneman and Riis, 2012). Especially, the teaching profession is a meaningful and influential work (Collie et al., 2015). For example, through music education, teachers can help students gradually establish correct values, philosophies, and healthy psychological quality, and can also cultivate students’ aesthetic interest, good quality, sense of honor, enterprising spirit, and intellectuality (Wang, 2011). Moreover, teachers with high well-being can maintain a better relationship with students, which is more conducive to stimulating students’ learning motivation and promoting students’ development (Anderson et al., 2004). In fact, many international studies have shown that more than one-third of teachers are under pressure or extreme pressure at work (Borg et al., 1991; Thomas et al., 2003; Geving, 2007). These worrying trends should make us think about teachers’ teaching well-being (Duckworth et al., 2009; Collie et al., 2012).

Teaching well-being is related to many factors. Erdogan et al. (2012) put forward the viewpoint of workplace well-being, that is, the level of individual well-being depends on satisfaction with the working environment, career development, leadership, and the fit between people and the environment. Other studies have pointed out that teachers’ well-being is largely affected by job stress and job burnout (Pakarinen et al., 2010; Spilt et al., 2011). Collie et al. (2015) specifically explained the factors that affect teachers’ well-being in the construction of teachers’ well-being model, including work pressure, organizational pressure, and student behavior-related pressure. Other scholars have also proved that these factors can have an important impact on teachers’ well-being (Aelterman et al., 2007; Konu et al., 2010).

Although research on teachers’ well-being has attracted more and more attention in recent years, most of them focus on non-workers such as patients, children, or adolescents (Joo and Lee, 2017), and there is still a lack of empirical research on the well-being of university teachers, especially university music teachers. Therefore, we believe that research on the well-being of university music teachers is an important gap. Moreover, Lyubomirsky et al. (2005) pointed out in the study that happy people are more likely to work actively toward new goals. Therefore, we can speculate whether university music teachers would have a better effect on teaching quality if they can maintain a sense of happiness in the teaching process. In addition, we also discussed the influence of flow experience on teaching well-being in this study. However, after consulting the relevant literature, we found that the experience of cardiac flow appeared more in the articles of physical exercise (Jackson, 1996; Srivastava and Mishra, 2015), e-learning environment (Esteban-Millat et al., 2014), and games (Chou and Ting, 2003; Klasen et al., 2012), which have relatively few applications in university classrooms. As an important factor in promoting well-being, it is necessary to introduce flow experience into teaching. Therefore, this study has important theoretical value to explore the relationship between university music teachers’ flow experience and teaching well-being through empirical research.

## Literature review and theoretical hypotheses

### Flow experience and well-being

The concept of flow was first proposed by Csikszentmihalyi (1991), an American psychologist. It refers to an immersive experience in which individuals devote themselves to an activity and show a high degree of excitement and satisfaction. In this state, individuals often experience a similar feeling of high concentration, automatically filter out ideas and thoughts unrelated to activities, loss of self-awareness, and strong control over the environment. Later, Beck (1992) summarized nine characteristics of flow in his article, namely, balance of challenge and technique, unity of behavior and consciousness, clear goals, full commitment, clear feedback, loss of self-consciousness, change of sense of time, contradiction of control, and self-directed sexual experience.

Teachers’ teaching well-being refers to teachers’ subjective psychological experience of all aspects of their profession, which is embodied in individuals’ positive evaluation of career motivation, work achievement, interpersonal relationships, and physical health (Van Horn et al., 2004). In terms of the investigation and research on teachers’ well-being, Dinham and Scott (2000) showed that teachers’ job satisfaction and well-being depend on their relationship with students, education and teaching methods, and care for students’ growth, while the requirement for social level is relatively low. Positive thoughts generated at work also help individuals enjoy a happier professional life (Raei Dehaghi, 2012). In addition, some scholars have indicated that factors such as work pressure and work achievement of teachers will also have an important impact on teaching well-being (Chapman, 2013). Therefore, we can attribute teachers’ teaching well-being to education itself rather than factors other than education.

To sum up, we propose the following hypothesis:

*H1*: University music teachers’ flow experience is significantly positively correlated with teaching well-being.

### Flow experience, work passion, and teaching well-being

Work passion means that employees are full of passion for their work, will devote a lot of time or energy, and will prioritize them, so as to achieve work goals as fun (Donahue et al., 2009). Lantara (2019) proposed that salary, working environment, leadership style, and their own education level will affect individuals’ work passion. In addition, some scholars have also studied the concept of work passion from the perspective of teachers, which refers to teachers’ passion for teaching or the discipline they teach, as well as teachers’ love for educators and students. Teachers’ work passion is also very important in the educational environment, which will affect teachers’ work attitude and behavior performance (Thayer-Bacon, 2004). For example, people who maintain a high degree of passion for work are often energetic, often try their best to achieve excellent results in work, and spend a long time on work without material rewards (Suliman, 2001). Wright (2007) also said that those individuals who work hard are not entirely for reward. What really supports them is intrinsic motivation, reward, and a sense of mission to work.

As a positive psychological theory, flow channel theory describes the psychological state of high concentration when individuals are immersed in a certain activity, which is characterized by concentration at work, without the need for external rewards, and a sense of problem-solving and innovation. It can generate a sense of satisfaction and pleasure, resulting in a sustained passion for the activity (Csikszentmihalyi, 1991). Especially when individuals encounter difficulties at work, this flow state will urge employees to regard overcoming pressure as a challenging task, which helps to stimulate employees’ passion for work, so as to deal with various problems (Lavigne et al., 2012). This passion is triggered by flow and external stimulation (Kaiser et al., 2012). Straume (2008) also said that individuals in the state of flow tend to have more positive work performance and obtain satisfaction from it. For example, the flow experience helps music teachers feel music with their hearts, integrate with music, express emotions with music, and have more musical creativity, so as to reach the peak (Sartika and Husna, 2014). In addition, it is worth noting that, according to the passion binary model of Vallerand et al. (2003), harmonious passions contribute to an individual’s job satisfaction, while compulsive passions may lead to individual tension, anxiety, and depression.

Emotional event theory shows that events workplace events trigger individual emotional responses, which in turn affect individual work attitudes and behaviors. Among them, the emotional meaning of events is an important factor leading to individual emotion-driven behavior (Weiss and Cropanzano, 1996). Therefore, we can assume that events that generate passion in individuals trigger emotional responses or actions that excite them (Frijda et al., 1989). Work passion, understood as an attitude and strong emotion in activity performance, has a significant impact on well-being (Bernabé et al., 2014) and can improve individual well-being (Vallerand et al., 2003). When individuals engage in activities that they are passionate about, they often experience positive effects (i.e., well-being or well-being) (Mageau and Vallerand, 2007). Gilal et al. (2019) said that teachers’ passion for work can be conveyed to students through emotion and other means. For example, teachers pass on the enjoyment of beauty to students through music, so that students can immerse themselves in it and achieve efficient learning. According to the theory of emotional events, teachers also experience pleasant emotions, known as well-being.

Therefore, we propose the following hypothesis:

*H2*: Work passion plays a mediating role in flow experience and teaching well-being.

*H2a*: Flow experience is positively correlated with work passion.

*H2b*: Work passion is positively correlated with teaching well-being.

### Flow experience, work engagement, and teaching well-being

Schaufeli et al. (2002) believed that work engagement refers to a positive and complete emotional and cognitive state related to work, which can maintain concentration without fatigue at work, and has the characteristics of persistence and dispersion, which is characterized by vigor, dedication, and absorption. Vitality means that an individual has plenty of toughness and energy, is not easy to be tired, and is willing to make continuous efforts in work. Dedication means that individuals have a strong sense of work engagement and can maintain a high degree of passion and pride in their work. Absorption is a state in which individuals are fully engaged in work (Schaufeli and Bakker, 2004). Teachers’ work engagement can be understood as a kind of working state in which teachers love and recognize educational work, and can actively participate in work and be tireless. Teachers’ work engagement has an important impact on teachers’ work efficiency and physical and mental health (Darling-Hammond and Youngs, 2002). For example, Hakanen et al. (2006) research shows that teachers with high work engagement are not prone to burnout and related health problems.

Self-determination Theory (Deci and Ryan, 1985) believes that human beings have natural interest and curiosity in new things, and can actively explore and learn. It is a spontaneous and innate essential feature. The behavior inspired by this internal motivation is called self-determined behavior (Deci and Ryan, 1985). As the term “flow experience” (Mirvis and Csikszentmihalyi, 1991) describes, individuals can completely immerse themselves in the work process and ignore what happens around them, and this experience is purely based on the support of internal motivation. Work engagement is also a positive state. People can concentrate on completing work tasks. However, flow is a peak experience based on current activities (Bakker, 2008), while work engagement is a long-term and lasting state (Hu and Wang, 2014). Some scholars have carried out research on the relationship between flow experience and engagement, and found that the process of flow experience is conducive to the formation of work engagement (Lovelace et al., 2007; Schueller and Seligman, 2010). Akiva et al. (2013) also showed that the production of cardiac flow can improve the level of individual work engagement.

Self-worth Theory (Covington, 1984) emphasizes that self-acceptance is the first need of people, and the premise of self-acceptance is to affirm self-worth. If individuals want to better realize their self-worth in the organization, they often set certain goals for themselves and drive themselves to work (Douglas et al., 2004). While individuals affirm and realize their self-worth, their teaching well-being index will also be improved to varying degrees (Covington and Beery, 1976). Waterman (1993) also believes that when individuals devote themselves to activities to give full play to their potential and realize their self-worth, individuals will reflect pleasure, that is, the feeling of well-being. In addition, a 7-year longitudinal study by Hakanen and Schaufeli (2012) found that the higher the degree of work engagement, the more conducive it is to reduce job burnout and other occupational stress problems, and thus improve personal well-being.

Therefore, we propose the following hypothesis:

*H3*: Work engagement plays a mediating role in the flow experience and teaching well-being.

*H3a*: Flow experience is positively correlated with work engagement.

*H3b*: Work engagement is positively correlated with teaching well-being.

### Work passion and work engagement

Work passion is an individual’s willingness to devote time and energy to work, which is a manifestation of attitude and behavior (Ho et al., 2011). It is typically characterized by confident, happy, motivated, and self-sustaining work (Wilson, 1993). Those who are passionate about their work feel meaningful no matter what they do (Hasanuddin and Sjahruddin, 2017). In addition, work passion is also a potential force, which can motivate individuals to participate in work spontaneously and generate positive behaviors (Chang, 2001). For example, in the education industry, teachers’ passion can have a positive impact on students’ performance and learning motivation (Kunter et al., 2013), thereby promoting the quality of teaching (Frenzel et al., 2009).

According to role investment theory, individuals devote their energy and time to roles that they find important and pleasant because this role provides them with a path to self-realization (Kanungo, 1979). As proposed by Bakker and Bal (2010), work passion can be regarded as an aspect of work engagement. When employees are passionate about their work, that passion can contribute to their success (Neumann, 2006). The attitude-behavior relational model (Ajzen and Fishbein, 1977) also proves that an individual’s attitude toward work ultimately drives individuals to exhibit positive work behaviors and good work outcomes. Other scholars have studied the effect of teachers’ work passion on work engagement, such as Frenzel et al. (2009) pointed out that teachers often feel passion with students, thereby increasing the sustainability of work engagement (Long and Hoy, 2006).

Therefore, we propose the following hypothesis:

*H4*: Work passion is positively correlated with work engagement.

**Figure 1:**
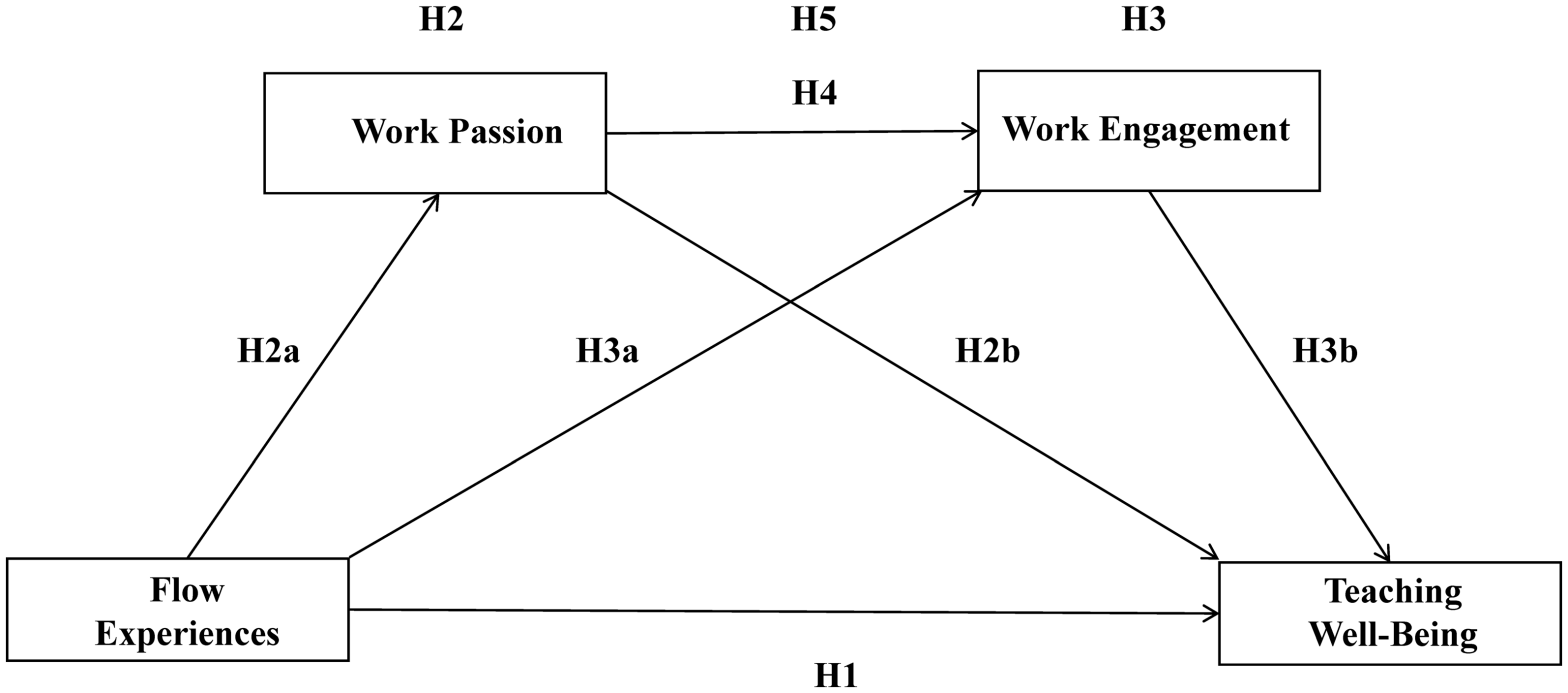
The conceptual model and research hypotheses of this study.

*H5*: Work passion and work engagement play a sequential mediating role between flow experience and teaching happiness. The conceptual model and research hypotheses of this study see Figure 1.

## Materials and methods

### Participants

This study used a cluster random sampling method. We first contacted the person in charge of the University Teacher Training Center of Shandong Province. Through him, we contacted 14 universities with music majors in Shandong Province and distributed electronic questionnaires. The survey was conducted in May 2022. Three hundred ninety-two teachers participated in this survey. For the returned questionnaires, 392 questionnaires were actually recovered, and the questionnaires that were obviously not in accordance with the normal time, missed, and incorrectly filled out were deleted, and finally, 343 valid questionnaires were obtained, with an effective recovery rate of 87.5%. See Table 1 for details. The study was ethically reviewed and approved according to the guidelines of the Declaration of Helsinki, and each faculty gave informed consent.

**Table 1:** Social demographic features of participants (*N* = 343).

Variables	Percentages
Gender	
Male	46.94%
Female	53.06%
Age	
30–32	56.56%
33–35	27.99%
36–38	6.12%
39–41	3.79%
42–44	5.54%
Title	
Teaching assistant	11.08%
Lecturer	72.30%
Associate professor	11.66%
Professor	4.96%

### Measures

#### Flow state scale

The scale is a nine-dimensional scale designed by Jackson et al. (2001) based on the nine components of the Nakamura and Csikszentmihalyi (2014) flow model, namely, challenge-skill balance, action-awareness merging, clear goals, unambiguous feedback, sense of control, and concentration on the task at hand, loss of self-consciousness, time transformation, and autotelic experience. The scale consists of 36 items, an example question is, “Often feeling that time flies.” The scale was assessed on a five-point Likert scale, ranging from 1 (strongly disagree) to 7 (strongly agree). In this study, Cronbach’s *α* was 0.962, and the Cronbach’s *α* values of dimensions were 0.851, 0.863, 0.876, 0.854, 0.877, 0.819, 0.856, 0.870, and 0.854, respectively.

#### Work passion scale

The Work Passion Scale (Vallerand et al., 2003) consists of two 7-item subscales: obsessive passion and harmonious passion. An example question is, “This activity is in harmony with other activities in my life.” The scale uses a 7-point Likert scale 1 (strongly disagree) to 7 (strongly agree) to assess teachers’ work passion. In this study, Cronbach’s *α* was 0.944, and Cronbach’s *α* values of dimensions were 0.939 and 0.940, respectively.

#### Work engagement scale

The study was assessed using the work engagement scale developed by Schaufeli et al. (2002). The scale is divided into three subscales: vitality, dedication, and focus. An example question is, “I feel that the work I do is purposeful and meaningful.” The scale is scored on a 7-point Likert scale ranging from (0 = strongly disagree) to (6 = strongly agree), with higher scores indicating a higher degree of teacher engagement. In this study, Cronbach’s *α* was 0.948, and Cronbach’s *α* values of dimensions were 0.938, 0.925, and 0.917, respectively.

#### Teacher well-being scale

The scale developed by Collie (2014) was used, which consisted of 16 items related to teachers’ work experience. The scale measures three factors of teacher well-being: organizational well-being, workload well-being, and student interaction well-being sense. An example question is, “Relations with students in my class.” The scale uses a 7-point Likert scale (1 = strongly disagree, 7 = strongly agree) to assess the well-being of different aspects of a teacher’s job. In this study, Cronbach’s *α* was 0.937, and the Cronbach’s *α* values of dimensions were 0.941, 0.943, and 0.921, respectively.

### Statistical methods and analysis ideas

In this study, SPSS 22.0 and Mplus version 8.3 were used for data analysis. SPSS was mainly used for data sorting, descriptive statistical analysis, etc. Mplus is mainly used for model inspection. Participants who lacked descriptive data or had many data points were treated by list deletion when running the analysis. In the analysis, teachers’ gender, age, and title were used as control variables. Gender is dummy coded (0 = female, 1 = male).

## Results

### Test of common method deviation

Using Harman’s single-factor test method, 15 factors with characteristic root greater than 1 were obtained. The explanation rate of the first factor is 30.152%, which is less than the cut-off value of 40% (Podsakoff et al., 2003), indicating that there is no significant common method bias in this study.

### Descriptive statistical analysis

Table 2 lists the major variables and Pearson correlation coefficients between each dimension. As can be seen from Table 2, all dimensions of teaching well-being were significantly positively correlated with all dimensions of flow experiences, all dimensions of work passion, and all dimensions of work engagement. According to the views of Tsui et al. (1995), in this study, the correlation coefficient of all variables is less than 0.75, and there is no serious multicollinearity problem among the major variables.

**Table 2:** Means, standard deviations, and correlations of the major study variables.

Variable	M	SD	1	2	3	4	5	6	7	8	9	10	11	12	13	14	15	16	17	18	19	20
1. Gender	0.469	0.500	1																			
2. Age	33.430	2.977	0.03	1																		
3. Title	2.100	0.645	0.055	0.872**	1																	
4. CSB	3.478	0.827	−0.021	0.034	0.033	1																
5. AAM	3.421	0.860	0.006	0.038	0.037	0.559**	1															
6. CG	3.442	0.878	−0.002	0.06	0.062	0.539**	0.559**	1														
7. UFB	3.322	0.877	−0.007	0.053	0.081	0.488**	0.434**	0.520**	1													
8. COTTAH	3.436	0.880	0.004	−0.045	−0.005	0.532**	0.559**	0.591**	0.557**	1												
9. SOC	3.665	0.788	−0.027	0.063	0.087	0.481**	0.475**	0.551**	0.463**	0.537**	1											
10. LOCS	3.343	0.875	−0.013	0.032	0.036	0.562**	0.564**	0.604**	0.546**	0.632**	0.545**	1										
11. TT	3.447	0.826	0.002	−0.018	−0.007	0.582**	0.542**	0.637**	0.572**	0.634**	0.561**	0.672**	1									
12. AE	3.568	0.826	−0.019	−0.029	−0.015	0.600**	0.591**	0.626**	0.535**	0.632**	0.508**	0.626**	0.619**	1								
13. HP	4.107	1.344	−0.013	0.018	0.021	0.323**	0.305**	0.323**	0.348**	0.383**	0.323**	0.342**	0.330**	0.362**	1							
14. AO	4.257	1.324	−0.061	−0.013	−0.02	0.325**	0.284**	0.285**	0.277**	0.335**	0.244**	0.298**	0.273**	0.362**	0.583**	1						
15. VI	3.176	1.391	0.016	0.032	0.022	0.303**	0.222**	0.264**	0.286**	0.337**	0.284**	0.315**	0.312**	0.270**	0.305**	0.283**	1					
16. DE	3.745	1.409	−0.001	0.059	0.083	0.286**	0.240**	0.254**	0.224**	0.344**	0.281**	0.338**	0.294**	0.268**	0.403**	0.360**	0.624**	1				
17. AB	3.331	1.321	−0.023	0.037	0.023	0.257**	0.220**	0.245**	0.241**	0.298**	0.277**	0.309**	0.295**	0.279**	0.311**	0.287**	0.586**	0.568**	1			
18. WWB	4.126	1.392	−0.011	−0.151**	−0.106*	0.179**	0.145**	0.275**	0.330**	0.346**	0.264**	0.303**	0.274**	0.302**	0.384**	0.342**	0.360**	0.379**	0.314**	1		
19. OWB	4.195	1.428	0.042	−0.206**	−0.134*	0.316**	0.364**	0.144**	0.219**	0.337**	0.280**	0.318**	0.301**	0.352**	0.350**	0.332**	0.349**	0.360**	0.314**	0.478**	1	
20. SIWB	3.827	1.514	0.019	−0.118*	−0.09	0.358**	0.419**	0.399**	0.351**	0.264**	0.190**	0.389**	0.378**	0.436**	0.299**	0.318**	0.313**	0.355**	0.321**	0.455**	0.506**	1

*N* = 343. **p < 0.01 and *p < 0.05. Gender is the dummy variable (0 = female, 1 = male). CSB, challenge-skill balance; AAM, action-awareness merging; CG, cear goals; UFB, unambiguous feedback; COTTAH, concentration on the task at hand; SOC, sense of control; LOCS, loss of self-consciousness; TT, time transformation; AE, autotelic experience; HP, harmonious passion; AO, assessing obsessive; VI, vitality; DE, dedication; AB, absorbed; WWB, workload well-being; OWB, organizational well-being; SIWB, student interaction well-being.

### Model inspection

The model was fitted by Mplus, the fitting index of the model was ML *χ*^2^ = 262.571, df = 155, *χ*^2^/df = 1.694, CFI = 0.963, TFI = 0.955, RMSEA = 0.045, SRMR = 0.035. Each index is in an acceptable range, and the model is ideal. See Table 3.

**Table 3:** Fit indices of the model.

Fit indices	Recommended threshold	Scores	Remarks
ML *χ*^2^	–	262.571	–
Df	–	155	–
*χ*^2^/df	1 < *χ*^2^/df < 3	1.694	Acceptable
CFI	>0.9	0.963	Acceptable
TLI	>0.9	0.955	Acceptable
RMSEA	<0.08	0.045	Acceptable
SRMR	<0.08	0.035	Acceptable

### The significance test of mediating effect

On the basis of good model fitting, the Bootstrap program of Mplus was used to repeat the sample for 5,000 times. The results show that the path coefficients of flow experiences, work passion, work engagement, and teaching well-being are all significant.

Flow experiences are positively related to teaching well-being (*β* = 0.248, *p* < 0.001), supporting H1. Flow experiences are positively related to work passion (*β* = 0.554, p < 0.001), supporting H2a. Work passion is positively related to teaching well-being (*β* = 0.295, *p* = 0.002), supporting H2b. Flow experiences are positively related to work engagement (*β* = 0.250, p = 0.002), supporting H3a. Work engagement is positively related to teaching well-being (*β* = 0.366, p < 0.001), supporting H3b. Work passion is positively related to work engagement (*β* = 0.419, p < 0.001), supporting H4. See Table 4.

**Table 4:** The direct effect of the research paths and research model hypothesis analysis.

DV	IV	Std. est.	SE	Est./SE	*P*-value	*R* ^2^	Hypo and path	Remarks
TWB	FE	0.248	0.070	3.551	***	0.635	H1: FE → TWB	Support
	WP	0.295	0.094	3.130	0.002		H2b: WP → TWB	Support
	WE	0.366	0.077	4.752	***		H3b: WE → TWB	Support
WP	FE	0.554	0.055	10.131	***	0.308	H2a: FE → WP	Support
WE	FE	0.250	0.080	3.116	0.002	0.356	H3a: FE → WE	Support
	WP	0.419	0.085	4.900	***		H4: WP → WE	Support

***p < 0.001. FE, flow experiences; WP, work passion; WE, work engagement; TWB, teaching well-being.

Table 5 shows the indirect effects of the study path. Work passion mediates the relationship between flow experiences and teaching well-being (*β* = 0.257, *p* = 0.005), with a 95% confidence interval [0.103–0.469], excluding 0, supporting H2, and the mediating effect accounted for 27.75%.

**Table 5:** The indirect effect of the research paths.

Path	Std. est.	SE	Est./SE	*p*-value	Boot LLCI	Boot ULCI	The proportion of the effect (%)
H2: FE → WP → TWB	0.257	0.091	2.819	0.005	0.103	0.469	27.75
H3: FE → WE → TWB	0.144	0.061	2.366	0.018	0.051	0.300	15.55
H5: FE → WP → WE → TWB	0.134	0.042	3.193	0.001	0.072	0.247	14.47
TOTALIND	0.536	0.101	5.277	***	0.363	0.779	57.88
TOTAL	0.926	0.123	7.536	***	0.711	1.189	100.00

*** *p* < 0.001.

Work engagement mediates the relationship between flow experiences and teaching well-being (*β* = 0.144, *p* = 0.018), with a 95% confidence interval [0.051–0.300], excluding 0, supporting H3, and the mediating effect accounted for 15.55%.

**Figure 2:**
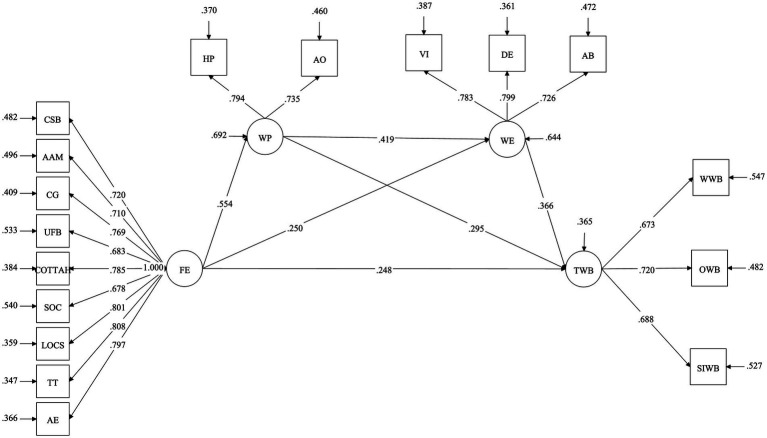
Structural equation model.

Work passion and work engagement sequentially mediate the relationship between flow experiences and teaching well-being (*β* = 0.134, *p* = 0.001), with a 95% confidence interval [0.072–0.247], excluding 0, supporting H5, and the mediating effect accounted for 14.47%. See Figure 2.

## Discussion

This study found that flow experience can significantly and positively predict teaching well-being, which is consistent with the research hypothesis and previous research results (O’Cass and Carlson, 2010; van Noort et al., 2012). The research also further verifies the relevant views of the positive emotion expansion theory (Fredrickson, 1998), which believed that when an individual is affected by a certain stimulus or meet their own needs, they will produce pleasant and positive emotions, which can keep the physiological function of the emotional subject happy and energetic. Like the profession of a music teacher, it includes not only an educational function but a profession that can make others and yourself better. Because music education is not only a kind of work, but also a kind of beautiful enjoyment. It is easy for music teachers to immerse themselves in their work and gain the experience of flow, which results in positive emotional experiences such as pleasure and well-being.

The results of this study show that work passion plays a mediating role between flow experience and teaching well-being. Flow experience can affect teaching well-being through work passion, that is, the higher the level of flow, the stronger the work passion, and the higher the level of teaching well-being. This conclusion verifies the relevant views of the flow channel theory (Mirvis and Csikszentmihalyi, 1991) and the emotional event theory (Weiss and Cropanzano, 1996). Music can bring pleasant feelings to people with its unique artistic charm, just as music teachers often need to demonstrate music skills to students with both voice and emotion in the teaching process, which can trigger teachers’ positive emotional experience, stimulate the flow experience, promote their work passion, and sprout the well-being of teaching.

In addition, this study also found that work engagement plays a mediating role between flow experience and teaching well-being. Flow experience can affect teaching well-being through work engagement, that is, the higher the flow experience, the stronger the work engagement, and the higher the level of teaching well-being. This conclusion validates the related views of self-determination theory (Deci and Ryan, 1985) and self-worth theory (Covington, 1984). For music teachers with a high degree of self-determination, they will actively teach and take teaching as a way to realize their own value, so as to gain a sense of teaching well-being. At the same time, they are more willing to actively participate in their work, and then experience the sense of success of self-worth realization. Self-actualization needs are the highest human needs. In the process of music teaching, music teachers’ self-realization needs are satisfied and self-worth is realized, and they should obtain a more sustainable teaching happiness experience.

In addition to finding that work passion and work engagement play a mediating role in flow experience and teaching well-being, respectively, this study also found that work passion and work engagement play a sequential mediating role in flow experience and teaching well-being. This also further validates the related views of role investing theory (Kanungo, 1979). A university music teacher who loves music education and students, has high work passion, is willing to actively invest time and energy in education, is willing to learn and innovate continuously, makes unremitting efforts to realize self-worth, and constantly strives to gain a sense of well-being.

This study also brings us relevant practical enlightenment. Firstly, it is necessary to improve the flow experience of music teachers and obtain teaching well-being. On the one hand, music teachers should maintain their love for music education. Just like “online shopping,” when we do something we like, we can forget time, get a flow experience, and experience the feeling of well-being. On the other hand, music teachers should be good at using technology to empower their work, and use multimedia technology to present teaching content, which is conducive to promoting the development of music teaching more vividly and truly immersed in teaching work. For example, in music appreciation, teachers can use multimedia technology to present teaching content, so that students will obtain psychological pleasure and aesthetic enjoyment through the emotional experience of music and art works (Fang, 2021). Teachers will also gain a sense of achievement and well-being in music teaching in the process. Secondly, enhance the passion of music teachers and improve the well-being of teaching. Organizations such as universities or related education and teaching departments can conduct relevant lectures and trainings for music teachers through various channels, increase music teachers’ understanding and learning of related content or courses, and stimulate music teachers’ passion for teaching. Finally, improve the level of work engagement music teachers and enhance well-being. Universities should create a relaxed working environment for music teachers, strengthen humanistic care, help teachers relieve pressure, allow music teachers to devote more energy to teaching and research, improve music teachers’ work engagement, and then improve their sense of well-being.

## Limitations and future research directions

Firstly, the data for this study are from self-report, which may be subjective. In the future, research, observation, interview, and other methods can be used to supplement the self-report method to improve the credibility of the results. Secondly, this study uses a cross-sectional study to explore the effect of flow experience on music teachers’ teaching well-being. Although the study is based on sufficient theory and empirical reasoning, and uses high reliability and validity measurement tools to analyze the data, the cross-sectional study cannot reflect the long-term performance of the mechanism studied in this study. Therefore, future research can consider using longitudinal research methods for more in-depth research. In addition, the data collected in this study are susceptible to the force majeure of COVID-19. Comparative studies could be considered in the future after COVID-19 is over or well controlled. Finally, this study only considers the chain intermediary role of work passion and work engagement in the flow experience and teaching well-being, and there may be other intermediary variables. Future research can consider the impact of other factors on music teachers’ teaching well-being. Moreover, potential feedback loops should also eventually be taken into account in future research. At the same time, we should notice that flow is amoral (Nakamura and Csikszentmihalyi, 2014), it is not good in an absolute sense (Mirvis and Csikszentmihalyi, 1991; Delle Fave et al., 2011), it has a dark side (Partington et al., 2009), and can also lead to persistence detrimental to psychological health (Chou and Ting, 2003). Therefore, in future research, the mediating effect of flow on passion and its risk of rigid persistence capable of damaging the psychological health of teachers needs to be considered.

## Conclusion

The results of this study show that there is a positive correlation between flow experience and teaching well-being. Flow experience can not only affect teaching well-being through work passion, but also affect teaching well-being through work engagement. In addition, this study also found that work passion and work engagement play a sequential mediating role between university music teachers’ flow experience and teaching well-being. We believe that these findings will provide an important basis for our future research on university music teachers’ teaching well-being and university management practice.

## Data availability statement

The raw data supporting the conclusions of this article will be made available by the authors, without undue reservation.

## Ethics statement

The studies involving human participants were reviewed and approved by the Research Ethics Committee of the Nanjing Normal University Taizhou College. The patients/participants provided their written informed consent to participate in this study.

## Author contributions

XW designed, prepared, and performed the data collection process, analyzed and validated the data, and wrote the article.

## Conflict of interest

The author declares that the research was conducted in the absence of any commercial or financial relationships that could be construed as a potential conflict of interest.

## Publisher’s note

All claims expressed in this article are solely those of the authors and do not necessarily represent those of their affiliated organizations, or those of the publisher, the editors and the reviewers. Any product that may be evaluated in this article, or claim that may be made by its manufacturer, is not guaranteed or endorsed by the publisher.
